# Biochemical Studies in Fibroblasts to Interpret Variants of Unknown Significance in the *ABCD1* Gene

**DOI:** 10.3390/genes12121930

**Published:** 2021-11-30

**Authors:** Stephanie I. W. van de Stadt, Petra A. W. Mooyer, Inge M. E. Dijkstra, Conny J. M. Dekker, Divya Vats, Moin Vera, Maura R. Z. Ruzhnikov, Keith van Haren, Nelson Tang, Klaas Koop, Michel A. Willemsen, Joannie Hui, Frédéric M. Vaz, Merel S. Ebberink, Marc Engelen, Stephan Kemp, Sacha Ferdinandusse

**Affiliations:** 1 Department of Pediatric Neurology, Emma Children’s Hospital, Amsterdam University Medical Centers, Amsterdam Neuroscience, University of Amsterdam, 1105 AZ Amsterdam, The Netherlands; s.i.vandestadt@amsterdamumc.nl (S.I.W.v.d.S.); m.engelen@amsterdamumc.nl (M.E.); 2 Laboratory Genetic Metabolic Diseases, Departments of Clinical Chemistry and Pediatrics, Amsterdam University Medical Centers, Amsterdam Gastroenterology Endocrinology Metabolism, University of Amsterdam, 1105 AZ Amsterdam, The Netherlands; p.mooyer@amsterdamumc.nl (P.A.W.M.); i.m.dijkstra@amsterdamumc.nl (I.M.E.D.); c.j.dekker@amsterdamumc.nl (C.J.M.D.); f.m.vaz@amsterdamumc.nl (F.M.V.); m.s.ebberink@amsterdamumc.nl (M.S.E.); s.ferdinandusse@amsterdamumc.nl (S.F.); 3 Regional Metabolic Clinic, Department of Medical Genetics, Southern California Permanente Medical Group, Los Angeles, CA 90027, USA; DIVYA.VATS@kp.org (D.V.); MOIN.U.VERA@kp.org (M.V.); 4 Departments of Neurology and Neurological Sciences and Pediatrics, Stanford, CA 94305, USA; ruzhnikov@stanford.edu (M.R.Z.R.); kpv@stanford.edu (K.v.H.); 5 Department of Chemical Pathology, Li Ka Shing Institute of Health Sciences, The Chinese University of Hong Kong, Hong Kong, China; nelsontang@cuhk.edu.hk; 6 Department of Metabolic Diseases, Wilhelmina Children’s Hospital, University Medical Center Utrecht, 3584 EA Utrecht, The Netherlands; K.Koop@umcutrecht.nl; 7 Department of Pediatric Neurology, Radboud University Medical Centre, 6525 GA Nijmegen, The Netherlands; Michel.Willemsen@radboudumc.nl; 8 Department of Pediatrics & Adolescent Medicine, Hong Kong Children’s Hospital, Hong Kong, China; joanniehui@yahoo.com.hk

**Keywords:** peroxisomal disorder, fibroblasts, adrenoleukodystrophy, variants of unknown significance, newborn screening

## Abstract

Due to newborn screening for X-linked adrenoleukodystrophy (ALD), and the use of exome sequencing in clinical practice, the detection of variants of unknown significance (VUS) in the *ABCD1* gene is increasing. In these cases, functional tests in fibroblasts may help to classify a variant as (likely) benign or pathogenic. We sought to establish reference ranges for these tests in ALD patients and control subjects with the aim of helping to determine the pathogenicity of VUS in *ABCD1*. Fibroblasts from 36 male patients with confirmed ALD, 26 healthy control subjects and 17 individuals without a family history of ALD, all with an uncertain clinical diagnosis and a VUS identified in *ABCD1*, were included. We performed a combination of tests: (i) a test for very-long-chain fatty acids (VLCFA) levels, (ii) a D_3_-C22:0 loading test to study the VLCFA metabolism and (iii) immunoblotting for ALD protein. All ALD patient fibroblasts had elevated VLCFA levels and a reduced peroxisomal ß-oxidation capacity (as measured by the D_3_-C16:0/D_3_-C22:0 ratio in the D_3_-C22:0 loading test) compared to the control subjects. Of the VUS cases, the VLCFA metabolism was not significantly impaired (most test results were within the reference range) in 6/17, the VLCFA metabolism was significantly impaired (most test results were within/near the ALD range) in 9/17 and a definite conclusion could not be drawn in 2/17 of the cases. Biochemical studies in fibroblasts provided clearly defined reference and disease ranges for the VLCFA metabolism. In 15/17 (88%) VUS we were able to classify the variant as being likely benign or pathogenic. This is of great clinical importance as new variants will be detected.

## 1. Introduction

The most common peroxisomal disorder, X-linked adrenoleukodystrophy (ALD), is characterized by mutations in the *ABCD1* gene [1,2]. Pathological mutations cause a dysfunction of the ALD protein (ALDP) [3], resulting in the impaired peroxisomal ß-oxidation of very-long-chain fatty acids (VLCFA; ≥C22:0) [4]. As a consequence, VLCFAs accumulate in plasma and tissues; primarily in the adrenal cortex, brain white matter and spinal cord [2,5]. Established pathogenic variants have no prognostic value with respect to clinical outcomes, and even monozygotic twins can have a vastly different disease course [6]. Clinically, all adult male patients and 80% of female patients develop symptoms of a slowly progressive myelopathy [7,8,9]. Furthermore, male patients—especially <12 years of age—are at risk of developing adrenal insufficiency and/or a progressive leukodystrophy (cerebral ALD) [10,11,12,13]. When diagnosed at an early stage, the treatment of cerebral ALD is possible with hematopoietic stem-cell transplantation (HCT) [14]. The adrenal insufficiency can be treated with a steroid replacement therapy. Due to these clinical implications, frequent follow-ups are advised for boys and men with ALD [10,15].

The availability of life-saving treatments and a better prognosis with an early diagnosis provided a rationale for including ALD in newborn screening (NBS) programs [16,17]. NBS is different from confirmatory diagnostic testing based on clinical suspicion. Due to the very low a priori probability of disease with NBS, even diagnostic tests with a very high specificity and sensitivity will result in false-positives and false-negatives [18]. In practice, this means that if a NBS is positive, but detects a variant of unknown significance (VUS) in *ABCD1*, there is a heightened level of diagnostic uncertainty. Clinical evaluation (which should be normal in a newborn with ALD) and repeated plasma VLCFA or C26:0-lysophosphatidylcholine (C26:0-lysoPC) testing can be helpful, but some uncertainty may remain. This is especially common for patients without a family history of disease or plasma VLCFA levels that are elevated between the upper limit of the reference range but below the lower limit of the disease range—here referred to as the “gray zone”. In these cases, additional biochemical and functional testing in skin fibroblasts may help to determine the pathogenicity of a VUS. These tests include: (1) the measurement of VLCFA levels; (2) a D_3_-C22:0 loading test (used to measure the D_3_-C26:0 de novo synthesis and D_3_-C22:0 ß-oxidation) [19]; and (3) immunoblotting for ALDP. The Genetic Metabolic Diseases (GMD) laboratory at the Amsterdam UMC is the only center worldwide that performs this combination of testing. With the increasing number of patients identified via newborn screening or exome/whole genome sequencing, we receive an increasing number of skin fibroblasts in individuals with a VUS in *ABCD1*—thus potentially suffering from ALD—from all over the world. For these tests to have a better predictive value, well-defined reference and ALD ranges are crucial, but not yet available.

The aim of this study was to establish the ranges for biochemical and functional tests in fibroblasts in ALD patients, as well as healthy controls to help with the interpretation of the results for patients with a VUS in *ABCD1*. To this end, we performed the aforementioned three tests on fibroblasts from control subjects and a large cohort of adult ALD patients. In addition, we included fibroblasts from 17 individuals without a family history of ALD, or with an otherwise uncertain diagnosis, in whom NBS or whole exome sequencing had identified a VUS in *ABCD1*. This study will contribute to a clearer and more uniform laboratory diagnostic process for ALD.

## 2. Materials and Methods

### 2.1. Study Design and Patient Selection (AMC, The Netherlands)

The present study is part of an ongoing observational cohort study on the national history of ALD—called the “Dutch ALD cohort”—performed at the Amsterdam University Medical Centers, which is located within the AMC, the national referral center for ALD. Participants visit the hospital yearly for multiple assessments including blood tests and an extensive neurological examination. For this particular cross-sectional study, we included male patients over 16 years of age with a confirmed ALD diagnosis (confirmed by an elevated VLCFA (C26:0, C26:0/C22:0 and C26:0-lysoPC) level and the presence of a pathogenic *ABCD1* mutation (Table 1)). Written informed consent was received from each patient. The study protocol was approved by the local Institutional Review Board (METC 2018–013). The fibroblasts of healthy controls subjects were collected for research purposes after the informed consent and the approval of the local Institutional Review Board.

### 2.2. Clinical Assessments of the Dutch ALD Cohort

Clinical assessments and skin biopsies took place on the same day. History and neurological examinations were performed as described previously [7]. We used the following clinical outcome measures to quantify the disease’s severity: the Expanded Disability Status Scale (EDSS), Severity Score System for Progressive Myelopathy (SSPROM) and Timed Up-and-Go (TUG). The EDSS measures neurological disability and ranges from 0 (no disability) to 10 (death) [20]. The SSPROM scores symptoms of myelopathy and ranges from 0 to 100, with lower scores indicating a higher degree of impairment [21]. TUG measures the time to get up from a chair, walk 3 m, walk back and sit down again [22].

### 2.3. ABCD1 VUS Fibroblasts

Fibroblasts from 17 individuals without a family history of ALD, but with a VUS in *ABCD1* were sent to our laboratory for diagnostic testing of the VLCFA metabolism from different (inter)national laboratories. Two of the *ABCD1* VUS in this cohort were eventually reported as “likely pathogenic” [23,24]. In this paper we name all (inter)national received fibroblasts ‘VUS’, as they were all initially reported as a VUS at the time of diagnostic testing. For all individuals, their clinical information, family history and follow-up biochemical results were obtained.

### 2.4. Biochemical and Functional Tests of VLCFA Metabolism

Fibroblasts were cultured in parallel in Ham’s F-10 Medium supplemented with 10% fetal bovine serum (Invitrogen, Carlsbad, CA, USA), 25 mM of HEPES, 100 U/mL of penicillin, 100 µg/mL of streptomycin and 250 µg/mL of amphotericin in a humidified atmosphere of 5% CO2 at 37 °C.

Total levels of VLCFA (C22:0, C24:0 and C26:0) in fibroblast homogenates were measured using gas chromatography–mass spectrometry essentially as has been described for plasma [25], but with the sample preparation described in Dacremont et al. [26].

The D_3_-C22:0 loading test was performed essentially as described in van de Beek et al. [19]. In short, cultured skin fibroblasts were incubated for three days with 30 µM of D_3_-C22:0 followed by a fatty acid analysis using electrospray ionization mass spectrometry [27]. The D_3_-C16:0/D_3_-C22:0 ratio, which is a measure of the peroxisomal ß-oxidation capacity, and D_3_-C26:0 levels, which reflect the de novo C26:0 synthesis, were calculated. In our results, the peroxisomal ß-oxidation activity is expressed as a percentage of the mean activity in the control cells analyzed in the same experiment. All cell lines were analyzed in two independent experiments (experimental replicates) and the measurements within the experiment were performed in duplicate (technical duplicates). The mean value of the measurements was used for the analysis.

For the immunoblotting of ALDP, cells were harvested by trypsinization and homogenized by sonication in phosphate buffered saline. Homogenates were diluted in a sample buffer before 10–20 μg of total protein was loaded on to an SDS/10% polyacrylamide gel. Proteins were transferred on to nitrocellulose by semi-dry blotting, blocked with 4% normal goat serum/PBS/0.1% Tween and probed with the primary monoclonal antibody against human ALDP (ALD-1D6-As, Euromedex or 197013, Abcam, Cambridge, UK). IRDYE 800CW (LICOR Biosciences, Lincoln, NE, USA) goat anti-mouse IgG or IRDYE 800CW (LICOR Biosciences, Lincoln, NE, USA) goat anti-rabbit IgG were used as a secondary antibody. A visualization of the signal was performed using the Odyssey IR imaging system (LI-COR Biosciences, Lincoln, NE, USA) [28].

### 2.5. Statistical Analysis

Data were summarized as means ± standard deviations (SD) or medians with ranges, depending on the distribution of the data. Scatter dot plots were used for the visualization of results for the three different groups: control cell lines, VUS cases and ALD patients. Additionally, we described the ranges for ALD patients and control subjects, defined as the minimum and maximum value per group. The gray zone was defined as the area between the upper and lower limits of the ALD values and the upper and lower limits of the control cell lines. Correlations between clinical measures and laboratory results were calculated with Spearmans’ Rho correlation coefficient. P-values lower than 0.05 were considered statistically significant. GraphPad Prism 8.3.0 and IBM SPSS Statistics Version 26 were used for the data analysis.

## 3. Results

### 3.1. Patient Characteristics

Thirty-six ALD patients were included in the analysis. The patients’ characteristics are described in Table 1. The clinical characteristics are described in Table 2. In summary, scores on clinical measures indicated a moderate disease severity. The mean age of the patients was 41.8 ± 17.2 years.

We included 17 individuals with a VUS (labeled VUS01–VUS17) in *ABCD1* from 5 different centers. VUS01 and VUS09 have the same variant (p.Lys533Gln), but came from different centers. VUS03 and VUS08 are two siblings. All VUS were detected via newborn/family screening or WES, except for one individual who presented with a decline in cognitive and behavioral function. None of the individuals had symptoms of ALD and none had a family history of ALD. The median age of the VUS group was 1.0 years (with a range of 2 months to 34 years) (Table 3).

### 3.2. VLCFA Levels in Fibroblasts

**Figure 1:**
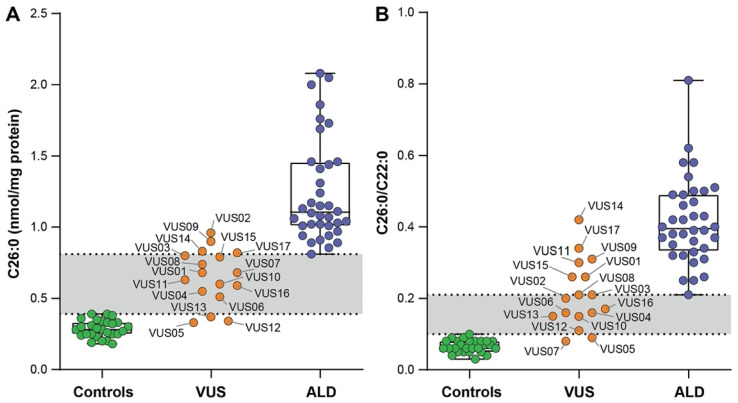
C26:0 levels (**A**) and C26:0/C22:0 ratios (**B**) analyzed using gas chromatography. Scatter dot plots show values for the fibroblasts of the control subjects (green circles); fibroblasts of the individuals with a VUS in *ABCD1* (orange circles); and fibroblasts of the ALD patients (blue circles). The VUS fibroblasts are labelled by ID. Both C26:0 levels and C26:0/C22:0 ratios were elevated in the ALD fibroblasts compared to control cells.

All ALD patient fibroblasts showed elevated levels of C26:0 (mean ± SD 1.25 ± 0.36 versus 0.29 ± 0.06 nmol/mg protein) when compared to the control cells (Figure 1A). In addition, the C26:0/C22:0 ratios were elevated in the fibroblasts of all ALD patients compared to the control cells (Figure 1B).

For the VUS cases C26:0 levels were within the reference range (0.18–0.39 nmol/mg protein) in 3 VUS (VUS 05, 12, 13) and elevated within the gray zone or ALD range in the remaining 14 (Figure 1A and Table 4). In addition, C26:0/C22:0 ratios were within the reference range (0.03–0.10) in 2 VUS (VUS 05, 07) and elevated in all other 15 (Figure 1B and Table 4).

### 3.3. D_3_-C22:0 Loading Test 

**Figure 2:**
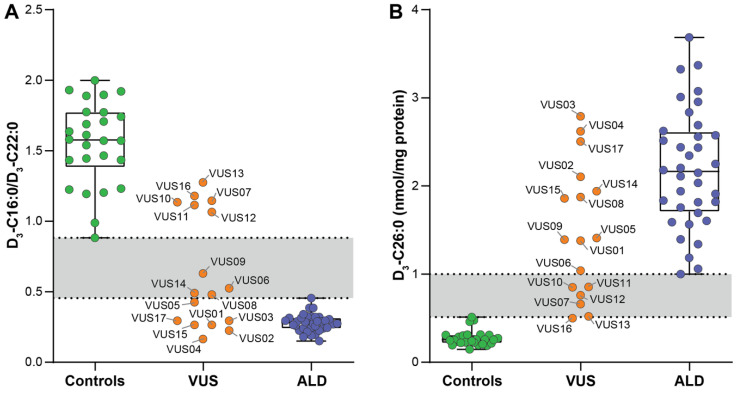
Results of the D_3_-C22:0 loading tests in the fibroblasts of the control subjects (green circles), fibroblasts of the individuals with a VUS in *ABCD1* (orange circles) and fibroblasts of the ALD patients (blue circles). The VUS fibroblasts are labelled by ID. (**A**) D_3_-C16:0/D_3_-C22:0 ratios, a measure of the peroxisomal ß-oxidation capacity, were reduced in the ALD fibroblasts compared to the control cells. (**B**) D_3_-C26:0 levels, reflecting chain-elongation, were elevated in the ALD fibroblasts compared to the control cells.

With the D_3_-C22:0 loading test the metabolism of D_3_-C22:0 in living cells can be followed, which is either elongated to D_3_-C26:0 or ß-oxidized to D_3_-C16:0. Hence, the ratio D_3_-C16:0/D_3_-C22:0 is a measure for peroxisomal ß-oxidation capacity. In all ALD patient fibroblasts the ratio of D_3_-C16:0/D_3_-C22:0 was reduced compared to the ratio in the control cells: the mean (±SD) ratio in the control cells was 1.55 ± 0.29 (range 0.88–2.00) and in ALD cells was 0.28 ± 0.06 (range 0.15–0.46) (Figure 2A). ALD fibroblasts had a median peroxisomal ß-oxidation activity in 17% of the controls measured in the same experiment (range 12–26%) (Table 4). Furthermore, chain elongation from D_3_-C22:0 to D_3_-C26:0 was increased in the ALD fibroblasts: the mean (±SD) D_3_-C26:0 in the control cells was 0.27 ± 0.09 (range 0.15–0.51) and in the ALD fibroblasts 2.19 ± 0.67 (range 1.00–3.69) (Figure 2B and Table 4).

In the VUS fibroblasts the D_3_-C16:0/D_3_-C22:0 ratio was reduced and within the ALD range (0.15–0.46) in 7 (VUS 01,02,03,04,05,15,17), reduced but in the gray zone (0.47–0.87) in 4 (VUS 06,08,09,14) and within the reference range (0.88–2.00) in 6 (VUS 07,10,11,12,13,16) (Figure 2A and Table 4). The peroxisomal ß-oxidation activity expressed as the percentage of the controls within the same experiments was reduced and within the ALD range (12–26%) in 7 (VUS01, VUS02, VUS03, VUS04, VUS14, VUS15 and VUS17), reduced but in the gray zone (26–56%) in 4 (VUS05, VUS06, VUS08 and VUS09) and within reference range in 6 (VUS07, VUS10, VUS11, VUS12, VUS13 and VUS16) (Table 4). Chain elongation to D_3_-C26:0 was increased and within the ALD range (1.00–3.69) in 11 (VUS01, VUS02, VUS03, VUS04, VUS05, VUS06, VUS08, VUS09, VUS14, VUS15 and VUS17), increased but in the gray zone (0.52–0.99) in 5 (VUS07, VUS10, VUS11, VUS12 and VUS13) and within the reference range (0.15–0.51) in 1 (VUS16) (Figure 2B and Table 4). The ALD and reference ranges for VLCFA metabolites and the D_3_-C22:0 loading test in the fibroblasts of this cohort are described in Table 4.

### 3.4. ALDP Immunoblot

In 27 out of 36 ALD patients (75%) immunoblotting for the ALD protein was negative, indicating that the variant in *ABCD1* resulted in no detectable ALD protein (Table 1). In the VUS group, immunoblotting was negative in two out of 17 (12%) variants: VUS01 and VUS09, which have the same variant, p.Lys533Gln. In all other cases, the ALDP protein was detectable, although the amount was reduced compared to the control levels in two (VUS02 and VUS04) (Table 4).

### 3.5. Clinical Correlations

**Figure 3:**
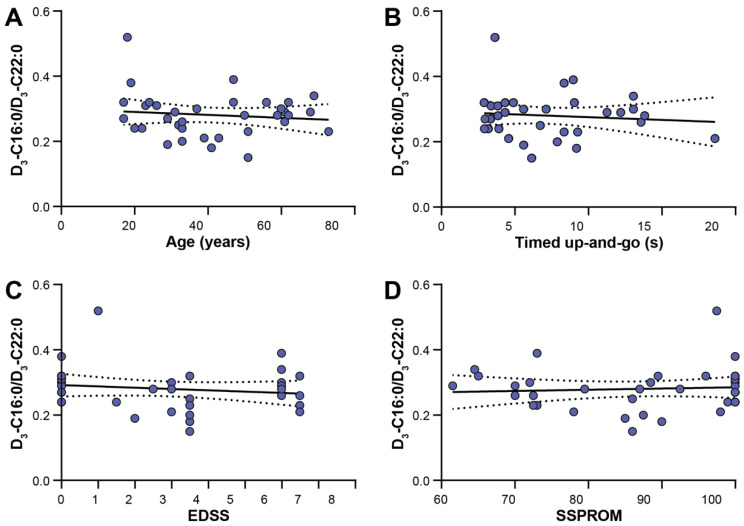
Scatter dot plots show the absence of correlations between clinical characteristics and residual peroxisomal ß-oxidation capacities (D_3_-C16:0/D_3_-C22:0 ratio). The lines represent simple linear regression lines with the 95% confidence interval. (**A**) Age, correlation coefficient −0.125, *p* = 0.467; (**B**) Timed Up-and-Go, correlation coefficient −0.092, *p* = 0.61; (**C**) EDSS (Expanded Disability Status Scale), correlation coefficient −0.173, *p* = 0.314; (**D**) SSPROM (Severity Score system for Progressive Myelopathy), correlation coefficient 0.197, *p* = 0.249.

For the 36 ALD patients, there were no associations (correlation coefficient ≤0.3 and *p*-value > 0.05) between measures for disease severity (Expanded Disability Status Scale (EDSS), Severity Score system for Progressive Myelopathy (SSPROM) and Timed Up-and-Go (TUG)), age nor residual peroxisomal ß-oxidation capacity (D_3_-C16:0/D_3_-C22:0 ratio) (Figure 3).

## 4. Discussion

This study shows that biochemical and functional studies in fibroblasts can provide a clearly defined range of the VLCFA levels and metabolism in cell lines in ALD patients and control subjects. This helps to classify variants in the *ABCD1* gene as either (likely) pathogenic or benign. A combination of all the included tests (VLCFA measurement, the D_3_-C22:0 loading test and immunoblotting for ALDP) and follow-up measurements enabled us to make an ALD diagnosis unlikely or likely in 15/17 (88%) of VUS (see Table 4).

For 6 out of 17 VUS (VUS07, VUS10, VUS11, VUS12, VUS13 and VUS16), we concluded that the *ABCD1* variant is likely benign and an ALD diagnosis is unlikely. From these six VUS, three (VUS 07, VUS12 and VUS13) had C26:0 levels or C26:0/C22:0 ratios within the reference range; the other three VUS yielded results within the gray zone. Furthermore, the D_3_-C26:0 levels were only mildly elevated (within the gray zone for all but one (VUS16, with normal levels)) and the D_3_-C16:0/D_3_-C22:0 ratios were within the reference range for all. The presence of peroxisomal ß-oxidation activity as a percentage of the control cells was ≥57% (range 57–107%) and the ALDP was present on immunoblots for all VUS individuals (or not performed for one: VUS16). Hence, we can conclude that these six *ABCD1* variants cause only a very minor impairment of the VLCFA metabolism. In all six VUS, VLCFA levels were normalized at follow-up, with the exception of VUS13, which only had a slightly elevated C26:0/C22:0 ratio. This strengthens our conclusion that these individuals are unlikely to develop clinical manifestations of ALD. However, one *ABCD1* variant (VUS11, c.1979G > A, p.Arg660Gln) has previously been reported as pathogenic in two patients with cerebral ALD and clearly elevated plasma VLCFA levels [24], and Western blotting showed an absence of ALDP in peripheral blood mononuclear cells from these patients [29]. In our cohort, the p.Arg660Gln was an incidental finding in a 5-year-old boy with another unrelated genetic condition. Because p.Arg660Gln was previously reported as pathogenic in the medical literature, there was uncertainty regarding the clinical diagnosis. In this boy (with VUS11), however, repeat measurements consistently showed normal plasma VLCFA levels. In order to help clarify this puzzling finding, we were asked to perform functional studies of the patients’ fibroblasts. Interestingly, our results are not in line with the earlier reports, as the immunoblotting for the ALDP in this individual (VUS11) was positive. Moreover, peroxisomal ß-oxidation was present in 59% of the control cells, and the D_3_-C16:0/D_3_-C22:0 ratio was 1.12, which is within the reference range (0.88–2.00). We have no definitive explanation for the discrepancy between our findings and the earlier reports, and it is puzzling why p.Arg660Gln results in non-detectable ALDP levels in one study and a normal ALDP expression in our study. Interestingly, the two ALD patients reported by Shukla et al. [24] both had additional synonymous variants in exon 9 (c.1899C > T (p.Ser633Ser) and c.1950G > A (p.Ala650Ala)). Synonymous variants in protein-coding regions are considered neutral for a protein’s function, as they synonymously exchange only codons and not the encoded amino acids. The ALD Mutation Database lists 120 synonymous variants in *ABCD1* (https://adrenoleukodystrophy.info/mutations-and-variants-in-abcd1, 28 October 2021). Although the effect of synonymous variants in *ABCD1* has not been investigated, we cannot rule out that they may affect the mRNA stability and ALDP expression, as has been described in the cases of other proteins [30]. It cannot be excluded that c.1899C > T (p.Ser633Ser) and/or c.1950G > A (p.Ala650Ala) aggravate the deleterious effect of p.Arg660Gln on ALDP expression or stability. Therefore, the presence or absence of such a functionally synonymous variant may determine the pathogenic outcome of a missense variant. Based on our extensive functional analyses we conclude that this p.Arg660Gln variant is likely benign.

In 9 out of 17 VUS (VUS01, VUS02, VUS03, VUS04, VUS08, VUS09, VUS14, VUS15 and VUS17), representing 7 different *ABCD1* variants (VUS03–VUS08 and VUS01–VUS09 carry the same variant), we concluded that the *ABCD1* variant is likely pathogenic and that an ALD diagnosis is likely. All of these VUS had elevated C26:0 levels and C26:0/C22:0 ratios (within the ALD range or gray zone). Results of the D_3_-C22:0 loading test were all within or very near the ALD range, with the peroxisomal ß-oxidation activity as a percentage of control cells ≤34% (range 13–34%). Immunoblotting for the ALDP was negative in two (VUS 01 and VUS09), reduced in two (VUS02 and VUS04) and positive in the remaining five VUS. These combined functional and ALDP expression data indicate that p.Lys533Gln (VUS 01 and VUS09), p.Thr483Met (VUS02) and p.Met566Val (VUS04) affect the protein folding and stability, resulting in no detectable or reduced levels of ALDP, whereas p.Lys610Glu (VUS03 and VUS08), p.Arg324Cys (VUS14), p.Ala643Thr (VUS15) and p.Asn192Asp (VUS17) affect amino acid residues that are important for ALDP function. Furthermore, plasma VLCFA levels were still elevated at follow-up in seven out of nine VUS; in one (VUS01) these results were not yet available. For VUS08 and VUS09, results of the D_3_-C22:0 loading test were within the gray zone, albeit close to the ALD range (peroxisomal ß-oxidation activity of 34%). However, for VUS01—with the same *ABCD1* variant as VUS09—and VUS03—which is a sibling (with the same *ABCD1* variant) of VUS08—results were well within ALD range. Based on these combined results, we conclude that the peroxisomal ß-oxidation activity as the percentage of control cells of 34% or less should be considered as highly suspicious with regard to ALD, even though the established ALD range in the studied cohort was 12–26%.

Of the two remaining VUS (VUS05 and VUS06), VUS05 had a peroxisomal ß-oxidation activity of 32%. However, the VLCFA analysis of fibroblasts was repeatedly normal, ALDP was normally expressed and plasma VLCFA was normal at follow-up. For VUS06, almost all results were within or close to the gray zone. Hence, we were unable to classify the *ABCD1* variants VUS05 and VUS06 with certainty. As more cell lines are analyzed, and remaining VUS are classified also based on long-term follow-up, the established ranges might need to be adjusted. Currently, it is not known if individuals with peroxisomal ß-oxidation activity within the gray zone will remain healthy or develop symptoms late in life. For instance, it is conceivable that an individual with slightly reduced ß-oxidation activity and slightly elevated VLCFA levels could develop symptoms, for example, a peripheral neuropathy, in (very late) adulthood. Therefore, these patients can be considered for inclusion in follow-up care, albeit with a low frequency, to monitor if or when symptoms will occur.

The lack of correlation between clinical disability and the D_3_-C16:0/D_3_-C22:0 ratio in fibroblasts indicates that the residual ß-oxidation capacity has no effect on the disease severity in ALD patients. Accordingly, one of our most severely affected patients—a 62-year-old male with an EDSS of 6.5—had a D_3_-C16:0/D_3_-C22:0 ratio of 0.32, which is relatively high for an ALD patient. These findings agree with previous research, in which a correlation between the VLCFA in plasma and disease severity was not found [31,32]. A certain threshold of accumulated VLCFA may need to be crossed in order to cause symptoms of ALD, but variation above this threshold is not of clinical importance. Furthermore, it is conceivable that metabolic studies in skin fibroblasts could be very useful for confirming the diagnosis, but that these parameters do not reflect the VLCFA metabolism in the central nervous system.

Our results should be interpreted with some caution. First, we defined the reference and ALD ranges in terms of the minimum and maximum values of each group. We chose these definitions instead of the mean +/− 2SD, because otherwise the ranges for the ALD and healthy control group would overlap. Moreover, not all test results followed a normal distribution, and we did not have sufficient statistical power to establish the ranges as mean +/− 2SD. However, we think that both groups are of sufficient size, considering the rareness of ALD, to determine the ranges as min–max, especially since we show a clear separation between both groups, with sometimes quite large gray zones in between. Furthermore, for the included VUS cases, a definite clinical diagnosis may not yet be possible, as they are young individuals who could be presymptomatic. Only a long term (many years or even decades) follow-up could provide a definitive diagnosis.

We can conclude that in this large cohort of ALD patients and control cell lines we could define clear ranges for the VLCFA metabolism in fibroblasts, which helped us classify 88% of the included VUS fibroblasts. We anticipate that an expansion of the cohort with additional cell lines from ALD patients would narrow the gray zone and further reduce the number of cases for which a conclusion of (likely) pathogenic or (likely) benign cannot be given. The availability of these functional test is of clinical importance, as the insecurity of a possible ALD diagnosis is a major burden for patients and their families. With the ongoing expansion of ALD NBS and increasing use of whole exome/genome sequencing in the routine diagnostic process, additional *ABCD1* variants will be detected. In case this results in the identification of a VUS, the array of functional tests described here will allow the confirmation or rejection of ALD diagnosis with more certainty, and hopefully will result in personalized clinical follow-up.

## Figures and Tables

**Table 1:** *ABCD1* variants, the effect on ALDP expression and VLCFA levels in fibroblasts of the ALD patients included in the study.

ID	*ABCD1* Variant	Consequence	ALDP Expression	C26:0	C26:0/C22:0
ALD022	c.1A > G	p.Met1Val	Not detectable	0.89	0.37
ALD030	c.1A > G	p.Met1Val	Not detectable	1.06	0.42
ALD043	c.1A > G	p.Met1Val	Not detectable	1.15	0.31
ALD062	c.1A > G	p.Met1Val	Not detectable	1.10	0.62
ALD063	c.1A > G	p.Met1Val	Not detectable	1.03	0.58
ALD064	c.1A > G	p.Met1Val	Not detectable	0.89	0.21
ALD031	c.220C > T	p.Arg74Trp	Not detectable	0.81	0.30
ALD056	c.411G > T	p.Trp137Cys	Not detectable	0.94	0.40
ALD034	c.446G > A	p.Ser149Asn	Detectable	1.08	0.37
ALD222	c.446G > A	p.Ser149Asn	Detectable	1.04	0.49
ALD003	c.529C > T	p.Gln177 *	Not detectable	1.15	0.25
ALD055	c.543C > A	p.Tyr181 *	Not detectable	1.01	0.39
ALD020	c.659T > C	p.Leu220Pro	Reduced	1.41	0.32
ALD045	c.734C > A	p.Ala245Asp	Detectable	1.02	0.49
ALD017	c.832G > T	p.Glu278 *	Not detectable	2.00	0.34
ALD228	c.874_76del	p.Glu292del	Not detectable	1.07	0.50
ALD058	c.892G > A	p.Gly298Ser	Detectable	1.17	0.43
ALD059	c.892G > A	p.Gly298Ser	Detectable	1.11	0.26
ALD081	c.892G > A	p.Gly298Ser	Detectable	0.91	0.81
ALD001	c.901-5C > A	p.Val301fs *?	Not detectable	1.86	0.43
ALD024	c.1166G > A	p.Arg389His	Reduced	0.97	0.38
ALD202	c.1166G > A	p.Arg389His	Reduced	1.24	0.33
ALD006	c.1322insA	p.Asp442Glyfs * 114	Not detectable	1.31	0.46
ALD011	c.1390C > T	p.Arg464 *	Not detectable	1.44	0.25
ALD899	c.1390C > T	p.Arg464 *	Not detectable	1.46	0.54
ALD009	c.1415_16delAG	p.Gln472Argfs * 83	Not detectable	2.08	0.35
ALD068	c.1488+3A > G	p.Val497fs *?	Not detectable	1.01	0.39
ALD069	c.1488+3A > G	p.Val497fs *?	Not detectable	1.01	0.39
ALD079	c.1846G > A	p.Ala616Thr	Not detectable	1.46	0.42
ALD025	c.1866-10G > A	p.Pro623fs *?	Not detectable	1.02	0.32
ALD048	c.1866-2A > T	p.Pro623fs *?	Not detectable	2.05	0.47
ALD049	c.1866-2A > T	p.Pro623fs *?	Not detectable	1.69	0.50
ALD027	c.1899delC	p.Ser633Argfs * 3	Not detectable	1.73	0.58
ALD008	c.1961T > C	p.Leu654Pro	Not detectable	1.76	0.51
ALD015	c.1961T > C	p.Leu654Pro	Not detectable	0.94	0.40
ALD019	c.1970delTCA	p.Ile657del	Not detectable	1.13	0.38

Reference ranges in fibroblasts for control subjects C26:0 (0.18–0.39 nmol/mg protein) and C26:0/C22:0 ratio (0.03–0.10).

**Table 2:** Clinical characteristics of ALD patients.

Clinical Characteristics	ALD Patients (*n* = 36)
Age (year)	41.8 ± 17.2
Body weight (kg)	80.4 ± 12.2
Adrenal insufficiency, n (%)	23 (64)
EDSS	3.0 (0.0–6.5)
SSPROM	89 (61.5–100)
TUG (s)	6.1 (2.9–18.6)

Values are displayed as the median (range) or mean ± SD. EDSS = Expanded Disability Status Scale; SSPROM = Severity Scoring System for Progressive Myelopathy; 6MWT = 6-min walk test; TUG = Timed Up-and-Go.

**Table 3:** Characteristics of individuals with a VUS in *ABCD1*.

ID	*ABCD1* Variant	Consequence	Method of Detection	Age	ALD Symptoms
VUS01	c.1597A > C	p.Lys533Gln ^b^	NBS	11 months	None
VUS02	c.1448C > T	p.Thr483Met	NBS	12 months	None
VUS03 ^a^	c.1828A > G	p.Lys610Glu	NBS	24 months	None
VUS04	c.1696A > G	p.Met566Val	NBS	5 months	None
VUS05	c.2134C > T	p.Arg712Cys	NBS	6 months	None
VUS06	c.566G > A	p.Arg189Gln	NBS	5 months	None
VUS07	c.895C > T	p.His299Tyr	NBS	2 years	None
VUS08 ^a^	c.1828A > G	p.Lys610Glu	Extended family screening	4 years	None
VUS09	c.1597A > C	p.Lys533Gln ^b^	NBS	2 months	None
VUS10	c.1000C > T	p.Leu334Phe	NBS	3 years	None
VUS11	c.1979G > A	p.Arg660Gln ^b^	WES	5 years	None
VUS12	c.896A > G	p.His299Arg	Decline in cognitive function	13 years	None
VUS13	c.1966_1967dup	p.Ile657ProfsX35 ^c^	NBS	2 months	None
VUS14	c.970C > T	p.Arg324Cys	NBS	2 months	None
VUS15	c.1900G > A	p.Ala643Thr	Family screening	34 years	None
VUS16	c.1438C > A	p.Pro480Thr	Family screening	9 years	None
VUS17	c.574A > G	p.Asn192Asp	NBS	2 months	None

^a^ Siblings; ^b^ mutation reported as likely pathogenic; ^c^ atypical zygosity. NBS = Newborn Screening; WES = Whole Exome Sequencing.

**Table 4:** VLCFA levels and D_3_-C22:0 loading test results of the VUS individuals compared to the ALD patients and control subjects.

Test	Reference Range	Gray Zone	ALD Range	VUS 17	VUS 14	VUS 02	VUS 03	VUS 01	VUS 04	VUS 15	VUS 09	VUS 08	VUS 05	VUS 06	VUS 07	VUS 10	VUS 11	VUS 12	VUS 13	VUS 16
C26:0	0.18–0.39	0.40–0.80	0.81–2.08	0.82	0.83	0.96	0.80	0.68	0.55	0.79	0.9	0.74	0.33	0.51	0.68	0.60	0.63	0.34	0.37	0.56
C26:0/C22:0	0.03–0.10	0.11–0.20	0.21–0.81	0.34	0.42	0.20	0.21	0.26	0.16	0.26	0.31	0.21	0.09	0.17	0.08	0.16	0.30	0.11	0.15	0.18
D_3_-C16:0	15.9–31.7	12.6–15.8	5.9–12.5	5.5	7.1	6.2	5.6	4.8	3.2	7.4	12.8	9.5	9.8	9.3	14.3	17.0	17.0	20.8	23.0	19.1
D_3_-C26:0	0.15–0.51	0.52–0.99	1.00–3.69	2.51	1.94	2.11	2.79	1.38	2.62	1.86	1.39	1.88	1.41	1.04	0.66	0.85	0.86	0.76	0.52	0.50
D_3_-C16:0/D_3_-C22:0	0.88–2.00	0.47–0.87	0.15–0.46	0.29	0.49	0.23	0.30	0.27	0.17	0.27	0.63	0.48	0.43	0.53	1.15	1.14	1.12	1.07	1.28	1.18
% of control cells	>57	27–56	12–26	13	25	17	19	23	14	18	34	34	32	43	67	57	59	107	72	61
Immunoblot				present	present	reduced	present	absent	reduced	present	absent	present	present	present	present	present	present	present	present	ND
VLCFA elevated at follow-up				Yes	Yes	Yes	Yes	ND	Yes	Yes	Yes	No	No	No	No	No	No	No	No	No

Green = within the control range; yellow = within the gray zone; red = within the ALD range. VUS01 and VUS09 have the same variant. VUS03 and VUS08 are siblings. ND = no data.

## Data Availability

Following publication, any data not published within this article will be shared by request from any qualified investigator.
